# Equilibrium and Dynamic Osmotic Behaviour of Aqueous Solutions with Varied Concentration at Constant and Variable Volume

**DOI:** 10.1155/2013/876897

**Published:** 2013-12-26

**Authors:** Ivan L. Minkov, Emil D. Manev, Svetla V. Sazdanova, Kiril H. Kolikov

**Affiliations:** ^1^Department of Chemistry and Biochemistry, Physiology and Pathophysiology, Faculty of Medicine, Sofia University, 1 Kozyak Street, 1407 Sofia, Bulgaria; ^2^Department of Physical Chemistry, Faculty of Chemistry and Pharmacy, Sofia University, 1 James Bourchier Boulevard, 1164 Sofia, Bulgaria; ^3^Faculty of Mathematics and Informatics, Plovdiv University, 24 Tzar Assen Street, 4000 Plovdiv, Bulgaria

## Abstract

Osmosis is essential for the living organisms. In biological systems the process usually occurs in confined volumes and may express specific features. The osmotic pressure in aqueous solutions was studied here experimentally as a function of solute concentration (0.05–0.5 M) in two different regimes: of constant and variable solution volume. Sucrose, a biologically active substance, was chosen as a reference solute for the complex tests. A custom made osmotic cell was used. A novel operative experimental approach, employing limited variation of the solution volume, was developed and applied for the purpose. The established equilibrium values of the osmotic pressure are in agreement with the theoretical expectations and do not exhibit any evident differences for both regimes. In contrast, the obtained kinetic dependences reveal striking divergence in the rates of the process at constant and varied solution volume for the respective solute concentrations. The rise of pressure is much faster at constant solution volume, while the solvent influx is many times greater in the regime of variable volume. The results obtained suggest a feasible mechanism for the way in which the living cells rapidly achieve osmotic equilibrium upon changes in the environment.

## 1. Introduction

Osmosis plays a primary role in biological systems. The exchange of matter with the medium in all living organisms occurs in such a mode. Osmosis is a physicochemical process, in which the concentration difference between two solutions creates pressure difference (*osmotic pressure*) across a separating semipermeable membrane. Solvent transport takes place from the more diluted solution to that of higher concentration, until equilibrium is reached. van't Hoff was the first [1] to propose a formula (*the van't Hoff law*) for the osmotic pressure *P*_osm_, exerted by the flow of solvent through the membrane:

(1) $$\begin{array}{c} P_{\text{osm}}=cRT, \end{array}$$

where *c* (mol/m^3^) is the molar concentration of the dissolved substance, *R* (8.314 J/mol K) is the universal gas constant, and *T* (K) is the absolute temperature. A number of more complex formulae for *P*_osm_ have been produced since [2–7]. Yet, in the course of the work, we have found the original van't Hoff law to be entirely sufficient for the particular tasks of our study.

Osmosis is in principle a process of diffusion, so it should be expected to obey the diffusion laws but its specific feature is that the species diffusing through the membrane are those of the solvent. Furthermore, in biological systems osmosis usually occurs in confined volumes, which may also impose its specificity. Krustev et al. [8] have introduced the term “*impeded osmosis*,” defined as “… osmosis at which practically constant solution volume is maintained by external mechanical influence, resulting in an increase of the hydrostatic pressure in this volume.”

For a long time since the discovery that the living cell membranes are built of lipid bilayers, it has been naturally assumed that water permeates these structures simply by diffusion. However, such diffusivity alone has proved utterly insufficient to explain the membrane permeability, as determined with real cell tissues [9]. Another track turned out to be much more dominant: the presence of protein channels of much higher capacity for water transport, such as aquaporins [10, 11]. For this reason, in the recent years, the discovery of the role of aquaporins as selective pores in water transport seems to have brought osmosis again into the focus not only of biological research but of technological practice as well.

Equilibrium studies of osmosis, whether theoretical or experimental, predominate in the literature. Nevertheless, the interest to the kinetic aspects of the process has never faded through the decades, in the past [12–15], as well as in the recent years [16–19].

Osmotic equilibrium is better understood from thermodynamic viewpoint and does not pose serious obstacles. On the other side, the dynamic aspects of the process frequently exhibit new and even surprising effects, which are difficult to explain within the frames of the traditional kinetic models.

The aim of our present investigation has been to look for possible specific effects by comparing the *P*_osm_ values in aqueous solutions under equilibrium and dynamic conditions as a function of solute concentration and time, while using an artificial semipermeable membrane and applying two different experimental regimes: of constant and variable solution volume. We believe that such a study of ours can produce results of relevance to the processes taking place in living matter and many technological applications.

In the classical membrane osmometry *P*_osm_ is directly determined by the hydrostatic pressure value established in an “open mode”—through the rise of the liquid level in the solution compartment. Of course, such an approach is only suitable at moderate elevation—of the order of decimeters—which, accordingly, means small concentration differences: up to a few tens of millimoles per liter (see (1)). An alternative mode, without such limitations, is conducting the process in a closed constant volume [3, 4, 7] and determining *P*_osm_ by means of an appropriate pressure sensor. The specific tasks of the present investigation required novel approach and modification of the classical experimental setup. Here we put forward an operative hybrid method, which combines the advantages of the two above: it comprises controlled variation of the solution volume, which permits measuring much higher pressure levels in the “open mode.”

In principle, the objectives of our study are the effects generated by the osmotic pressure in living organisms, where its magnitude is commonly limited to relatively low values, rarely exceeding fractions of one atmosphere. Still, in the regime of variable volume, we have extended the explored concentration range to 0.5 mol/L, in order to outline more clearly the established effects. For the complex tests we have chosen sucrose, a low molecular mass compound, repeatedly used as a reference in osmotic studies [3–6, 13].

## 2. Materials and Methods

High purity (Sigma-Aldrich 99+%) sucrose was employed in all experiments. Polyamide composite semipermeable Koch RO (reverse osmosis) membranes were used within the prescribed ranges (pH = 4–11; temperature <50°C). Our tests confirmed the assertion of Grattoni et al. [7] that sucrose is thus totally filtered. All solutions were prepared with Elga Labwater (model PURELAB Option-Q7) deionized water. The membrane osmometer employed in our experiments was specially designed and built for the purpose [20]. It consists of two cylindrical plastic shells, for solvent and solution, respectively. A semipermeable membrane of 5.0 cm diameter was sealed between the two shells and was supported against deformations by additional plastic porous disks on either side. The active operative area of the membrane (the integral hole surface) was ca. 5 cm^2^. Further details about the original device are discussed in the respective patent [20].

**Figure 1:**
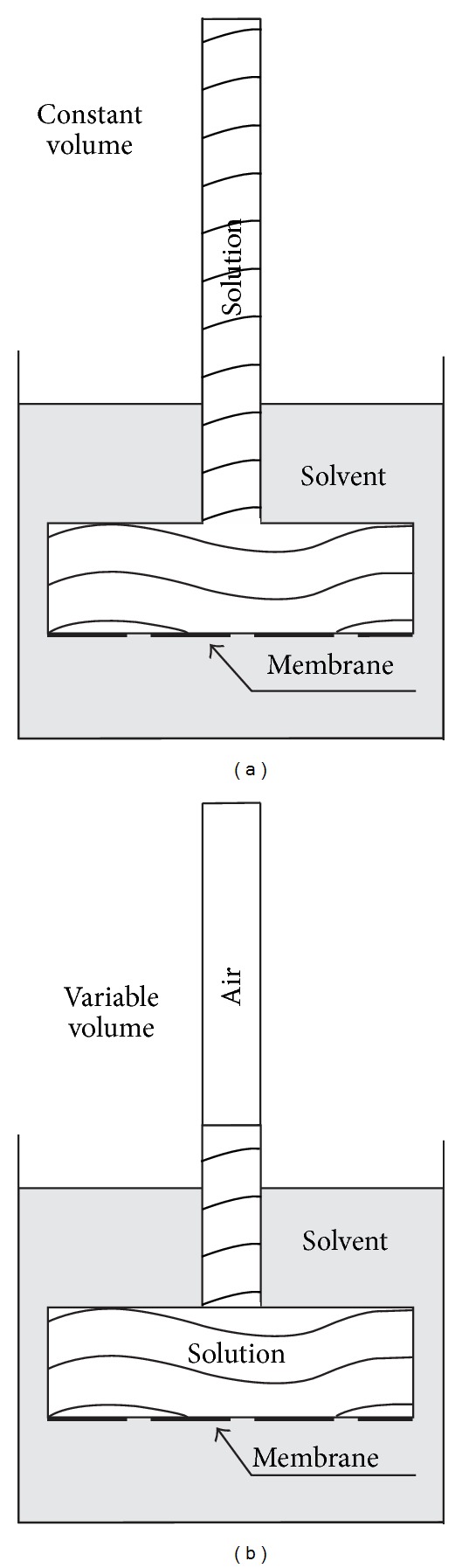
Schematic of the two experimental osmotic regimes: (a) open cell (variable volume); (b) closed cell (constant volume).

The specific tests, in particular the comparison between osmotic rates at free (variable) and restricted (constant) volume, required further modifications. Here comes in action our hybrid modification of the cell with limited variation of the solution amount by additional partial volume. Thin graduated 1.3 m long plastic tubing of 2 mm radius was attached to the solution chamber to measure the solution level rise at variable volume. Thus, with initial capacity of the solution compartment of 60 mL, the attached tube provided additional volume of 16.5 mL, that is, a possibility of variation by up to ca. 25%. We consider this sufficient for our present purpose. Of course, we could have supplied even larger span of volume variation, but such a step would have brought further complication, due to the substantial dilution of the studied solution upon time. Both versions of the measuring osmotic cell are presented schematically in Figure 1.

**Figure 2:**
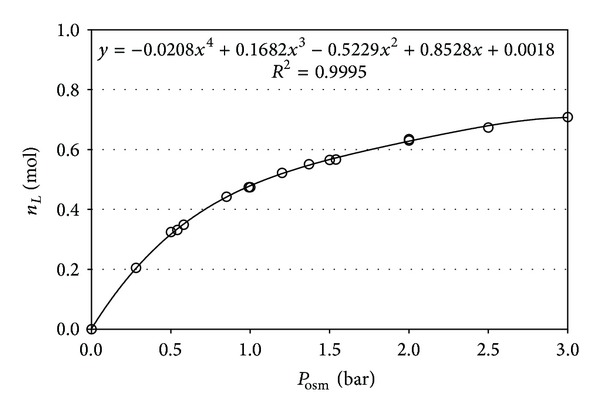
Solvent influx *n*_*L*_ [mol] as a function of osmotic pressure *P*_osm_ [bar] in the regime of variable solution volume.

Of course, as required by the gas laws, in the case of volume variation (*A*) the “solvent influx versus pressure” dependence is not linear; the flux steadily decreases upon building osmotic pressure, as illustrated by Figure 2. Yet, for the purpose of our comparison here such nonlinearity does not create any problems. The ultimate solution concentrations were derived by means of the amount of solvent passed through the membrane. The corresponding osmotic pressure is registered by a 16-bar electronic pressure sensor (reading ±0.01 bar). All experiments were conducted at a temperature of 22°C.

## 3. Results and Discussion

Experimental tests were designed and realized at constant and varied solution volume, comprising two targets.

### 3.1. Dependence of the Equilibrium Osmotic Pressure on the Solute Concentration

**Figure 3:**
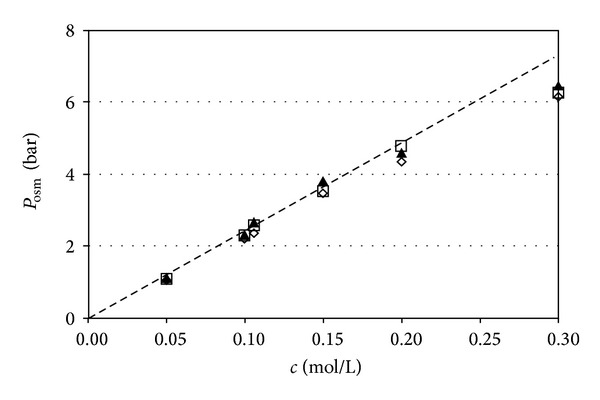
Comparison of equilibrium osmotic pressure values, *P*_osm_ as a function of solute concentration under regimes of constant and variable solution volume. Experimental data are from the readings of the electronic pressure sensor, respectively: (□) constant solution volume; (◊) variable solution volume; (▲) variable solution volume (values corrected for dilution). The dotted line indicates the theoretical dependence (see (1)).

The systematic measurements of the “*P*_osm_ versus *c*” dependence were performed with 5 solute concentrations in the range 0.05 M–0.30 mol/L. The results are presented in Figure 3 and Table 1.

Due to the transfer of solvent, in the process conducted with an open cell (variable solution volume) the concentration of the studied solution is reduced to a detectable extent. Hence, an attempt was made to introduce appropriate corrections on the basis of the known initial amount of solvent in the osmotic cell (ultimate column to the right of Table 1).

The results obtained for both regimes, as conducted, respectively, at constant and variable solution volumes, certainly indicate very close *P*_osm_ values, practically undistinguishable within the limits of accuracy of the experimental measurements. Further on, except for the highest studied concentration of 0.3 mol/L, the experimentally obtained values of *P*_osm_ lie very close to the dependence estimated from (1) and the scatter of measured values is within the range of the experimental error: ±1.1%, estimated in accordance with the theory in [21]. Computation with permitted inaccuracy of pressure determination of ±0.01 bar and of solute concentration of ±0.001 mol/L yields a maximal error for the osmotic pressure value, as determined in the studied range, of ±0.07 bar.

### 3.2. Comparison of the Osmotic Rate Values at Closed and Variable Cell Volume

**Figure 4:**
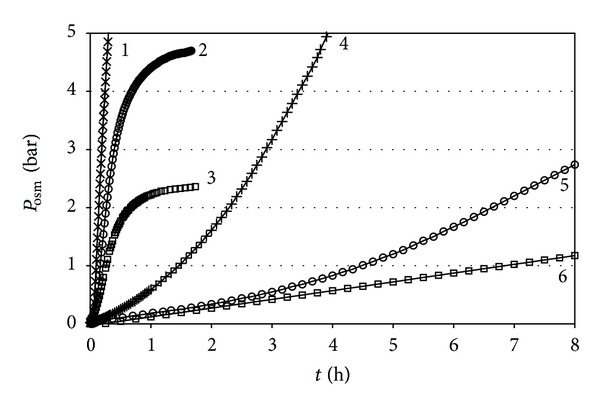
Osmotic pressure *P*_osm_ versus time *t* dependence for three different initial sucrose concentrations at the two regimes: (1) 0.5 M (constant volume); (2) 0.2 M (constant volume); (3) 0.1 M (constant volume); (4) 0.5 M (variable volume); (5) 0.2 M (variable volume); (6) 0.1 M (variable volume).

**Figure 5:**
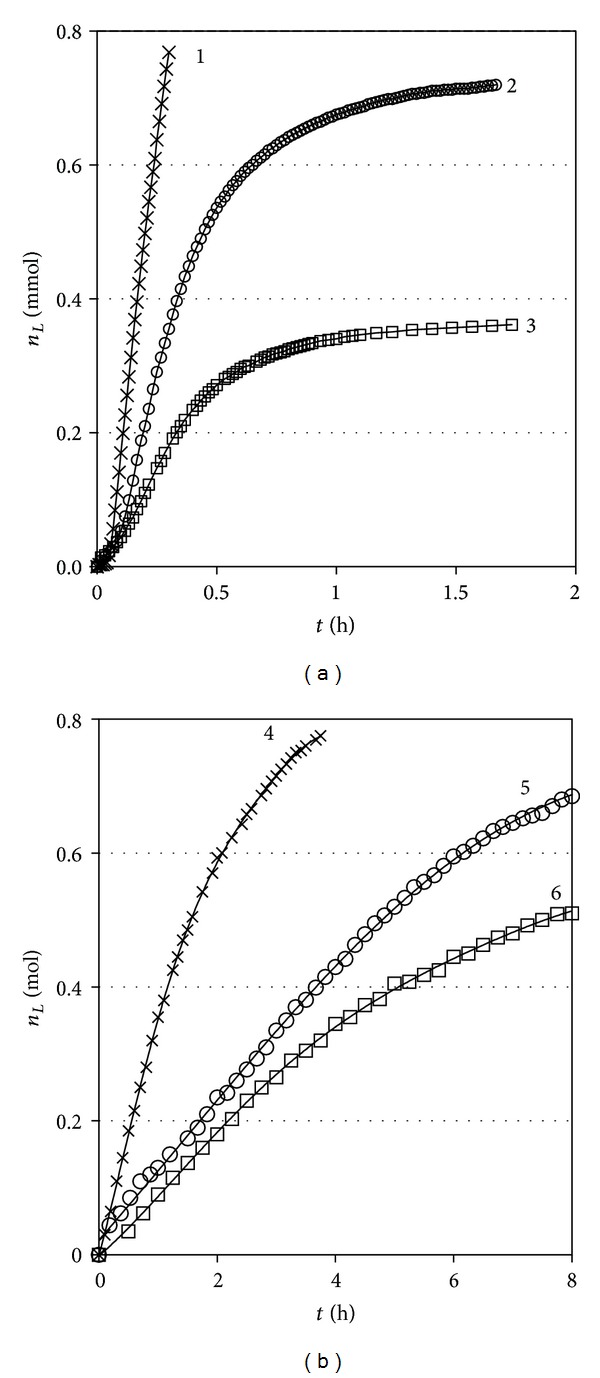
Solvent influx *n*_*L*_ as a function of elapsed time *t* dependences for the three studied solute concentrations: (a) constant volume regime: (1) 0.5 M; (2) 0.2 M; (3) 0.1 M (*n*_*L*_ is expressed in millimoles); (b) variable volume regime: (4) 0.5 M; (5) 0.2 M; (6) 0.1 M (*n*_*L*_ is expressed in moles).

**Figure 6:**
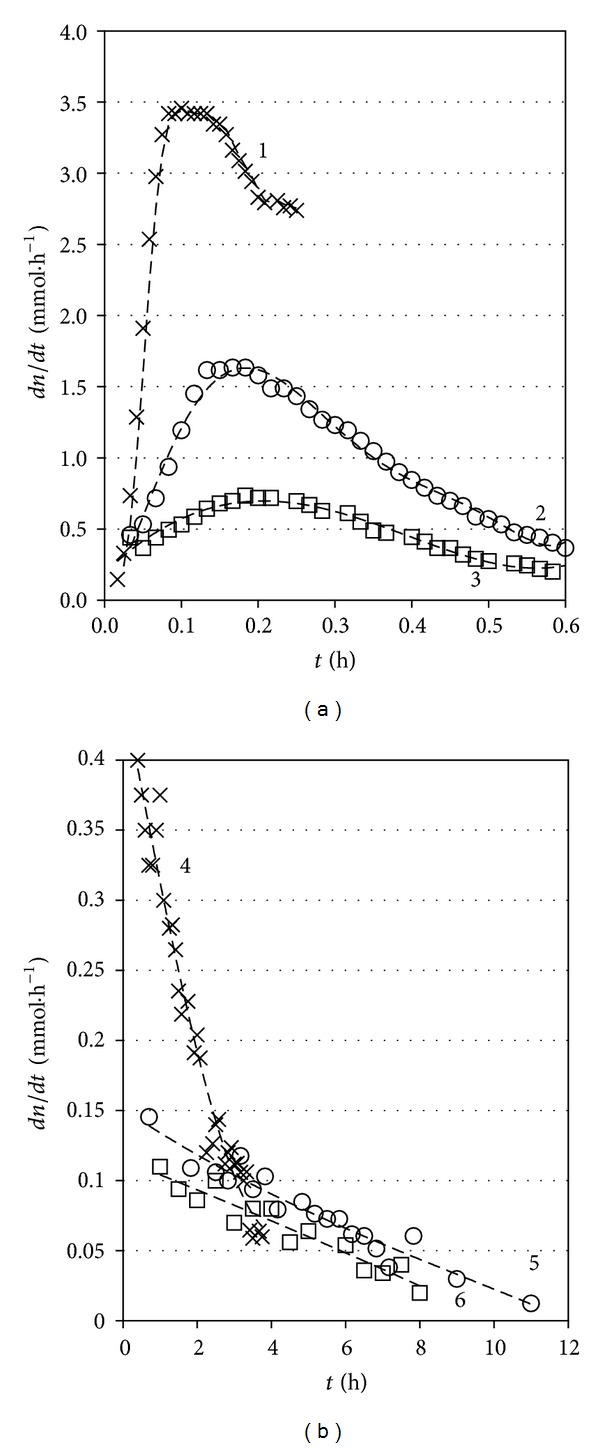
Solvent rates of transfer dependences *dn*_*L*_/*dt* as a function of elapsed time *t* for the three solute concentrations: (a) constant volume regime: (1) 0.5 M; (2) 0.2 M; (3) 0.1 M (*n*_*L*_ is expressed in millimoles); (b) variable volume regime: (4) 0.5 M; (5) 0.2 M; (6) 0.1 M (*n*_*L*_ is expressed in moles).

In the kinetic experiment three solute (sucrose) concentrations were chosen for the comparison of the osmotic rates for processes at constant and variable solution volume: 0.1 M, 0.2 M, and 0.5 M. The results are presented in Figures 4, 5, and 6 and Table 2.

The juxtaposition of the kinetic dependences presented in Figure 4 demonstrates the drastic differences in the rates of osmotic pressure rise for the two regimes. With variable cell volume, the osmotic pressure rise occurs at much slower rate. However surprising at first sight, this finding can be regarded as a quite natural result. The amount of solvent, which has to pass into the solution compartment of the cell, in order to lift the osmotic pressure, differs dramatically in the two regimes. For example, employing the value for the coefficient of compressibility of pure water of 4.6 × 10^−5^ bar^−1^, one estimates that for a closed cell of solution volume of 60 cm^3^ the amount of solvent needed to raise the pressure by one atmosphere is 2.76 × 10^−3^ cm^3^ ( = 1.53 × 10^−4^ moles H_2_O). Concurrently, in our case of limited solution volume variation by additional 16.5 cm^3^ to the initial of 60 cm^3^, the amount of solvent necessary to lift the pressure up to a level of *P*_osm_ = 1.0 bar will be ca. 8.5 cm^3^ ( = 0.47 moles of water; cf. Figure 2). The latter amount is some 3000 times (i.e., more than by three orders of magnitude) larger than that at constant volume and, of course, will definitely require longer time for transport.

For the sake of comparison we can also employ the classical case of unlimited solution volume variation. For an osmotic cell connected to an open tube of radius as small as 2 mm, the amount of solvent necessary to lift the solution level by 10.2 m (in order to impose hydrostatic pressure of 1 atmosphere) would be 4*π* × 10^−2^ (cm^2^) × 1.02 × 10^3^ (cm) = 128 cm^3^.

Further on, Figures 5(a) and 5(b) reveal another remarkable finding. While with variable solution volume the osmotic pressure rise, as shown in Figure 4, is always faster at constant volume and the flow through the membrane is much faster in the regime of variable volume. One must note that the ordinate axis scales of the two sections of Figure 5 differ by three orders of magnitude! Thus, the solvent influx rates at variable regime turn to be by two orders of magnitude larger in practically all studied cases.

One feature of interest in the kinetic behaviour of the studied systems in the two regimes is the different trends that the solvent transfer rates follow with time, as shown in Figures 6(a) and 6(b).

The osmotic process at constant volume appears to start at very slow rate for all concentrations, sharply accelerate with time, and pass through an expressed maximum. Then the rate of solvent transfer declines more gradually, eventually reaching values several times lower than those at the maxima. The amplitude depends on the solute (sucrose) concentration.

This result is surprising and far from easy to interpret. We would have rather expected fairly steady rates, especially in the initial stages, away from equilibrium. Yet, the initial increase may be attributed to a delayed response of the semipermeable membrane to the early impact of solvent, to which it needs time to adjust. The onset of the decline beyond the maximum appears to occur too early to be interpreted in terms of decreasing driving force of the osmotic process (the difference between equilibrium and instant osmotic pressure values) toward equilibrium. In all three cases the pressure values are still sufficiently far from the respective upper limit of *P*_osm_ (see further in Table 2).

The picture is different in the regime of varied solution volume. The solvent transfer rates in this case uniformly decrease with time at all three studied solution concentrations, but the dependence for the highest level of 0.5 M stands out in contrast to those for the lower concentrations of 0.2 and 0.1 M. A major factor in this case appears to be the nonlinear dependence of solvent transfer across the membrane toward the solution “per unit change of osmotic pressure” (cf. Figure 2). This nonlinear dependence would be mainly responsible for the experimentally registered continuous decline of solvent influx, down to 20–30% of the initial transfer rate, as the osmotic pressure rises to values of the order of 4 bar at the higher sucrose concentrations of 0.2 and 0.5 mol/L. At the lowest studied concentration of 0.1 mol/L, threefold decrease of the solvent influx rate is attained even at as low osmotic pressure as 1.25 bar. However, in the latter case the effect of approaching the osmotic equilibrium might have imposed certain influence. As it can be seen from Figures 3 and 4 and Table 1, the pressure limit we must expect there is 2.35 ± 0.07 bar.

Under the studied conditions another though a lesser factor appears to be also the concentration decrease of the solution in the progress of osmosis at variable solution volume, due to the larger influx of solvent into the additional space, appended to the solution compartment. This effect of dilution may account for some 10–15% of the total change (decrease) in the solvent transfer rates on the way to equilibrium.

These results are outlined in Table 2, which presents the osmotic characteristics, as estimated at the two different regimes for the three chosen sucrose concentrations: maximal rates of the osmotic pressure rise at constant volume (*dP*/*dt*)_const_ and variable volume (*dP*/*dt*)_varied_, the respective maximal values of the solvent influx rates (*dn*/*dt*)_const_ and (*dn*/*dt*)_varied_, the times registered for the maximal influx rates values (*τ*), and the ratios of instant to equilibrium pressure at the influx maxima. The second part of the table provides comparison of the ratios of the respective pressure and solvent influx rates at the two regimes. The solvents influx rates are computed using an estimated value for the active membrane area of 4.65 cm^3^.

We can summarize in brief the present findings as follows.

- The applied here novel approach of limited variation of solution volume has proved efficient and productive for the osmotic experiments.
- The obtained “pressure versus time” dependences reveal that the rise of pressure is much faster at constant solution volume for all studied solute concentrations.
- Inversely, the solvent influx through the semipermeable membrane toward the solution is many times greater in the regime of variable volume.
- The values of flow rate at constant solution volume pass through expressed maxima, while at variable volume they steadily decrease with time. The latter effect may be principally attributed to the applied technique of limited variation of solution volume. Concurrently, the dilution of the operative solution in the progress of the process can only account for a small fraction of the decrease.
- The results obtained suggest a feasible mechanism, applicable for the way in which the living cells rapidly achieve osmotic equilibrium upon changes in the environment, despite osmosis being considered as a slow process in general.

Summing up from a biological standpoint, we consider the established effects to be of relevance for the processes taking place in the living cells. The osmotic dynamics is of prime importance for the cellular homeostasis. On cellular level, the adaptation to external influences would not have been possible without such fast-occurring processes, as is the mass transfer, and the rapid attainment of osmotic equilibrium in confined volumes. The dynamics of the osmotic processes in the cell could not have been realized if the cellular volumes were not relatively small and only slightly varying in response to the environmental changes.

## 4. Conclusions

The initiated here study of aqueous solutions offers a new approach in the investigation of osmosis under different experimental conditions, comprising limited solution volume expansion as a tool to widen the scope of studied concentrations and pressures. The results, as obtained, demonstrate the applicability and the advantages of the new method when comparing the osmotic behaviour at constant and variable volume.

At equilibrium, the results certainly indicate very close values of the osmotic pressure for processes conducted at constant and variable solution volume, practically undistinguished within the limits of accuracy of the experimental measurements.

On the other hand, the kinetic rate values for the two regimes differ markedly. The fact that, at a given solute concentration, the pressure increase for constant solution volume occurs at much faster rate is a natural result, considering the vastly dissimilar amount of solvent transferred into the solution compartment. However, in terms of solvent flow rates, the picture displays the opposite: transfer of liquid is much faster in the case of variable volume. Moreover, the established patterns of pressure and liquid influx rates differ significantly as a function of time, so do most of the osmotic characteristics, as determined at the two regimes.

The results obtained in the current investigation have allowed our deriving convincing conclusions about the distinction in the kinetics of the osmotic process under different regimes: of constant and variable solution volume. The data generated by means of the new method are of relevance to understanding the mechanisms of self-maintaining the living cell homeostasis. Further on, in stricter quantitative terms, the interpretation of the obtained differences is much more complex and would demand additional considerations. This, however, is beyond the scope of the present investigation and is the subject of our next study.

## Figures and Tables

**Table 1:** Comparison of the osmotic pressure values at variable (open) and constant (closed) volume with the theoretical estimates.

Solute concentration *c *[mol/L]	*P*_osm_ [bar](1)	*P*_osm_ [bar] (constant)	*P*_osm_ [bar] (variable)	*P*_osm_ [bar] (variable; corrected)
0.050	1.21	1.10	1.06	1.11
0.100	2.42	2.29	2.20	2.30
0.106	2.57	2.57	2.36	2.66
0.150	3.64	3.51	3.46	3.81
0.200	4.84	4.77	4.35	4.58
0.300	7.27	6.26	6.14	6.45

**Table 2:** Comparison of the kinetic characteristics of the osmotic process in aqueous sucrose solutions for the two experimental regimes of constant and variable solution volume. Active area of the semipermeable membrane *S*_*M*_=4.65 cm^3^.

Studied solution (concentration/experimental regime)						
	0.1 mol/L		0.2 mol/L		0.5 mol/L	
	(Constant/varied)		(Constant/varied)		(Constant/varied)	
*dP*/*dt* [mbar/s]	1.306	0.044	2.94	0.15	6.22	0.52
*dn*/*dt* [mmol/m^2^s]	0.448	67.0	0.996	88.3	2.10	243.6
*τ* [s]	720	3600	570	2520	390	1440
*P*_*τ*_/*P*_eq_	0.107	0.016	0.194	0.029	0.298	0.050

	0.1 mol/L		0.2 mol/L		0.5 mol/L	

(*dP*/*dt*)_con,max⁡_ (*dP*/*dt*)_var,max⁡_	29.68		19.63		11.92	
(*dn*/*dt*)_var,max⁡_ (*dn*/*dt*)_con,max⁡_	149.5		88.68		115.7	
